# Comparative Assessment of In-House Real-Time PCRs Targeting Enteric Disease-Associated Microsporidia in Human Stool Samples

**DOI:** 10.3390/pathogens10060656

**Published:** 2021-05-26

**Authors:** Konstantin Tanida, Andreas Hahn, Kirsten Alexandra Eberhardt, Egbert Tannich, Olfert Landt, Simone Kann, Torsten Feldt, Fred Stephen Sarfo, Veronica Di Cristanziano, Hagen Frickmann, Ulrike Loderstädt

**Affiliations:** 1 Department of Microbiology and Hospital Hygiene, Bundeswehr Hospital Hamburg, 20359 Hamburg, Germany; konstantintanida@bundeswehr.org (K.T.); frickmann@bnitm.de (H.F.); 2 Department of Medical Microbiology, Virology and Hygiene, University Medicine Rostock, 18057 Rostock, Germany; Hahn.andreas@me.com; 3 Department of Tropical Medicine, Bernhard Nocht Institute for Tropical Medicine & I. Department of Medicine, University Medical Center Hamburg-Eppendorf, 20251 Hamburg, Germany; k.eberhardt@bnitm.de; 4 Institute for Transfusion Medicine, University Medical Center Hamburg-Eppendorf, 20251 Hamburg, Germany; 5 Bernhard Nocht Institute for Tropical Medicine Hamburg, 20359 Hamburg, Germany; tannich@bnitm.de; 6 National Reference Centre for Tropical Pathogens, 20359 Hamburg, Germany; 7 TIB MolBiol, 12103 Berlin, Germany; OLandt@tib-molbiol.de; 8 Medical Mission Institute, 97074 Würzburg, Germany; simone_kann@hotmail.com; 9 Department of Gastroenterology, Hepatology and Infectious Diseases, University Medical Center Düsseldorf, 40225 Düsseldorf, Germany; Torsten.Feldt@med.uni-duesseldorf.de; 10 Department of Medicine, Kwame Nkrumah University of Science and Technology, Kumasi, Ghana; stephensarfo78@gmail.com; 11 Institute of Virology, Faculty of Medicine and University Hospital of Cologne, University of Cologne, 50935 Cologne, Germany; veronica.di-cristanziano@uk-koeln.de; 12 Department of Hospital Hygiene & Infectious Diseases, University Medicine Göttingen, 37075 Göttingen, Germany

**Keywords:** microsporidiosis, real-time PCR, test comparison, *Enterocytozoon bieneusi*, *Encephalocytozoon* spp., stool, latent class analysis

## Abstract

Microsporidiosis is an infection predominantly occurring in immunosuppressed patients and infrequently also in travelers. This study was performed to comparatively evaluate the diagnostic accuracy of real-time PCR assays targeting microsporidia with etiological relevance in the stool of human patients in a latent class analysis-based test comparison without a reference standard with perfect accuracy. Thereby, two one-tube real-time PCR assays and two two-tube real-time PCR assays targeting *Enterocytozoon bieneusi* and *Encephalocytozoon* spp. were included in the assessment with reference stool material (20), stool samples from Ghanaian HIV-positive patients (903), and from travelers, migrants and Colombian indigenous people (416). Sensitivity of the assays ranged from 60.4% to 97.4% and specificity from 99.1% to 100% with substantial agreement according to Cohen’s kappa of 79.6%. Microsporidia DNA was detected in the reference material and the stool of the HIV patients but not in the stool of the travelers, migrants, and the Colombian indigenous people. Accuracy-adjusted prevalence was 5.8% (*n* = 78) for the study population as a whole. In conclusion, reliable detection of enteric disease-associated microsporidia in stool samples by real-time PCR could be demonstrated, but sensitivity between the compared microsporidia-specific real-time PCR assays varied.

## 1. Introduction

Microsporidiosis is a disease caused by intracellular pathogens related to fungi as indicated by phylogenetical analyses [1]. Although microsporidia were already known at the beginning of the 20th century [2], they had initially been misidentified as primitive protozoa and only later were assigned to the phylum of fungi [3], while the phylogenetic assignment remained controversial [4]. More recent assessments consider microsporidia as “intracellular parasites … related to fungi” [1,5]. Many important metabolic pathways are missing due to their extremely reduced and compacted genomes [6]. The genera *Nosema*, *Vittaforma*, *Brachiola*, *Pleistophora*, *Encephalitozoon*, *Enterocytozoon*, *Septata* (reclassified to *Encephalitozoon*), and *Trachipleistophora* have been associated with human disease [2]. Thereby, the four species *Enterocytozoon bieneusi*, *Encephalitozoon cuniculi, Encephalitozoon hellem,* and *Encephalitozoon intestinalis* are considered as the quantitatively most relevant ones with particular focus on *Enterocytozoon bieneusi* [7]. Therapeutic options are scarce and poorly standardized, comprising tubulin-inhibiting albendazole (*Encephalocytozoon* spp.) and methionine aminopeptidase type 2-inhibiting fumagillin or its less toxic derivates (*Enterocytozoon bieneusi*) [8,9].

Microsporidia-associated diarrhea, the most frequent clinical manifestation, is usually an opportunistic medical condition in immunocompromised individuals [10]. The causative agents are abundant worldwide but with particular emphasis on resource-limited settings like sub-Saharan Africa [11]. However, in industrialized countries like the People’s Republic of China, an infection rate about 8% has also recently been shown for *Enterocytozoon bieneusi* in the stool of patients with acquired immunodeficiency syndrome (AIDS) [12]. In Iran, intestinal infection rates of more than 10% have been described for immunosuppressed patients with diarrhea and particularly low CD4 cell counts of <200/µL [13]. The well-established zoonotic potential of microsporidia [7,9] as well as health risks for immunocompromised individuals due to enrichment of microsporidia via the food chain have been recently discussed [14].

Due to their likely etiological relevance in immunosuppressed individuals, microsporidia have also been demonstrated as a threat to patients under chemotherapy or immunosuppressive drugs, e.g., after transplantation [15]. Nevertheless, microsporidiosis can also infrequently occur in the immunocompetent host, but severely debilitating and even lethal courses are associated with profound immunosuppression [16,17]. However, among not obviously immunocompromised individuals like children, the elderly and travelers, microsporidia have also been detected with increased frequency, partly as asymptomatic colonization [18]. Asymptomatic, latent infections in individuals with intact T-cell mediate host defense have been shown by serological approaches [19].

While gastrointestinal disease with chronic diarrhea and wasting is the most frequent manifestation of clinically apparent microsporidiosis, focal disease like keratoconjunctivitis, cerebritis, or hepatitis has been described as well [16]. Transmission of the pathogens occurs in a spore stage which uses a polar tube as an invasion apparatus to invade the host cell [16]. Within the host cell, microsporidia affect the transcription of various genes involved in innate immunity, ubiquitylation, metabolism, or hormonal signaling and altered degradation pathways [20]. Further, the microbes efficiently harness the host’s metabolism to facilitate their development and reproduction [21].

Intestinal microsporidiosis can be diagnosed from stool samples or small bowel biopsies applying light microscopy with special stains or electron microscopy of the very small spores as well as molecular tools like real-time PCR [10,22,23]. Regarding traditional microscopy of stool samples on stained slides, especially the combination of chitin-staining fluorochromes like Calcofluor White and the modified trichrome stain, has been associated with acceptable sensitivity, immunofluorescence staining with acceptable specificity [24]. Immunofluorescence assays have also been applied for the identification of microsporidia spores in body fluids and tissues [25,26]. In tissues, alternatives to immunostaining comprise Gram stain and Giemsa stain [24].

In particular, ribosomal RNA sequences of microsporidia are frequently used for molecular diagnostic purposes [2,22,27]. Molecular assays targeting microsporidia are in routine use as cost-efficient alternative to microscopical approaches [28]. Accordingly, a variety of real-time PCR assays have been developed for the identification of the most frequently identified microsporidia in human samples, *Enterocytozoon bieneusi* and *Encephalocytozoon* spp., for diagnostic and surveillance purposes [29,30,31,32,33,34,35,36,37,38]. In this study, we have compared the diagnostic performance of different in-house real-time PCR assays for the identification of microsporidia in stool samples without a reference standard with perfect accuracy applying latent class analysis (LCA). Of note, LCA is an indirect method for diagnostic accuracy estimation. As a variant of structural equation models, this approach estimates non-observed variables as the disease status over observed variables, i.e., the results of diagnostic tests [39,40]. Stool samples of various populations that are known to show increased intestinal carriage of microsporidia [18] have been used for the assessment.

## 2. Results

Depending on the assay or assay combination applied, the number of positive samples within the assessed 1339 samples ranged from 47 (PCR 2) to 87 (PCR 1). Microsporidia-specific DNA was detected in diagnostic samples from the diagnostic department of the Bernhard Nocht Institute in Hamburg and in the samples from the HIV patients from Ghana only, while no microsporidia DNA was identified in stool of any other assessed population. Of note, there was no potentially interfering positivity of PCR 6 with specificity for the non-target fungal agents *Microsporidium* spp. observed; all samples remained negative in PCR 6.

When applying LCA testing for the estimation of test accuracy, the best sensitivity was recorded for PCR 1, followed by the assays PCR 4 + 5, PCR 3 + 5, and PCR 2 in descending order. With the exception of PCR 2, all assays showed a sensitivity of 95% or better, while the sensitivity of PCR 2 scored considerably poorer with a sensitivity of 60.4%. Focusing on specificity, no major differences were observed with a specificity >99% for all assays or assay combinations assessed.

Focusing on Cohen’s kappa, substantial agreement between the assessed assays could be demonstrated. With focus on media cycle threshold (Ct) values, PCR 1 showed 4–5 cycles earlier signals compared to the competitive assays (Table 1).

## 3. Discussion

The study was performed to assess the diagnostic accuracy of different published real-time PCR assays for the detection of important microsporidia with pathogenic potential for humans [33,35,37,38,41] in stool samples in a test comparison approach without a reference standard with perfect accuracy. Substantial agreement between the compared assays as well as good specificity of all assessed assays or assay combinations could be observed. Also, cross-reactivity with a real-time PCR for the non-target fungi *Microsporidium* spp., which are similar only by name but phylogenetically distinct, was excluded to make incidental interference due to non-target fungal DNA occurring in stool less likely. The comparably high limit of detection of the applied *Microsporidium* spp. PCR weakens the interpretability of this additional specificity control, an admitted limitation of the study.

Although PCR 1, which is also diagnostically applied at the Bernhard Nocht Institute for Tropical Medicine, Hamburg, for routine diagnostic purposes, scored best regarding sensitivity, it was in a range similar to the competitor combinations PCR 3 + 5 and PCR 4 + 5 of ≥95%. Only the SybrGreen assay PCR 2 showed a considerably poorer sensitivity of only 60.4%, so it is likely that microsporidia DNA may be overlooked when applying this approach. The observation of the reduced sensitivity of PCR 2 is in line with previous results from a test comparison using an alternatively composed sample population [36]. An obvious association between amplicon length and sensitivity of the assessed real-time PCR assays was not observed.

As a side effect, an overall prevalence of detected microsporidia DNA ranging between 3.5% (*n* = 47) and 6.5% (*n* = 87) of the samples was recorded for the assessed sample population. Upon applying the LCA to calculate test accuracy-adjusted prevalence [38,40], we obtained a prevalence of 5.8% (*n* = 78). Positive samples were only detected among the reference sample materials from the Bernhard Nocht Institute Hamburg and from the sub-population of the HIV-positive Ghanaian patients. The latter is well in line with the known association of microsporidiosis and immunosuppression [10]. In 416 included samples from travelers, migrants, and Colombian indigenous people as indicated in the methods section below, however, no DNA of microsporidia was detected, so the previously reported association with international travel [18] could not be confirmed by our study.

The study has a few limitations. First, due to a lack of availability of microscopically well-characterized samples, LCA as an indirect method for diagnostic accuracy estimation had to be applied. Second, restrictions associated with the ethical clearance for this test assessment did not allow the presentation of clinical data associated with the assessed samples other than the general descriptions as provided in the methods section. Third, a considerably low proportion of positive samples led to broad 95% confidence intervals for the sensitivity estimations. Fourth, considerable sample age of 10 years and more might have interfered with DNA quality, although the applied mode of DNA storage as detailed below is generally associated with excellent preservation in the authors’ experience.

## 4. Materials and Methods

### 4.1. Sample Materials

A total of 1339 residual nucleic acid extractions from stool samples were included in the assessment. Those materials comprised 20 residual samples from patients that had tested positive for intestinal microsporidiosis in the routine diagnostics department of the Bernhard Nocht Institute for Tropical Medicine in Hamburg (which is the German National Reference Center for Tropical Pathogens in Hamburg, Germany), 59 residual samples from a previous study in the Colombian tropics [42], 140 residual samples from migrants [43], 195 residual samples from German policemen deployed in the tropics [44], 22 residual samples from German soldiers [45] returning from tropical deployments, and 903 residual DNA samples from African HIV-positive patients from previous studies from Ghana [46,47]. The samples were chosen to ensure a sufficient likelihood of intestinal carriage of microsporidia as associated with the history of travel [18] or immunosuppression [10]. In line with the ethical clearance for this test comparison, the residual sample materials were anonymized and no patient-specific data could be presented, which would be an admitted violation of the STARD (Standards for Reporting Diagnostic Accuracy) criteria [48]. All samples had been stored at −80 °C prior to their submission for periods between few months and 15 years.

### 4.2. Nucleic Acid Extraction and Internal Amplification Control

Nucleic acids were extracted applying the QiaAMP DNA Stool Mini kit (Qiagen, Hilden, Germany) as suggested by the manufacturer. The extracted DNA was stored at −80 °C prior to the assessments. Internal amplification control was based upon a real-time PCR targeting a Phocid Herpes Virus (PhV) sequence as described elsewhere [49].

### 4.3. Applied Target-Specific PCRs

All real-time PCRs were run on magnetic induction cyclers (MIC; Bio Molecular Systems Ltd., London, UK). Positive controls consisting of target sequences within plasmids and negative controls consisting of PCR-grade water were included in each run. Two out of five target-specific real-time PCRs targeted both *Enterocytozoon bieneusi* as well as the three etiologically relevant *Encephalocytozoon* spp., *Encephalitozoon cuniculi, Encephalitozoon hellem,* and *Encephalitozoon intestinalis*.

Real-time PCR 1, targeting small subunit ribosomal RNA, was designed based on sequences from previous papers [38,41]. In particular, the two forward primers were identical with the primers MICRO-F (forward-1) and Msp2 (forward-2) from the work by Wang et al. [38] and amended by a common probe and four different reverse primers specifically targeting *Enterocytozoon bieneusi* (reverse-1, GenBank accession number: AF023245), *Encephalitozoon cuniculi* (reverse-2, GenBank accession number: MT483990.1), *Encephalitozoon hellem* (reverse-3, GenBank accession number: MG584868.1), *Encephalitozoon intestinalis* (reverse-4, GenBank accession number: CP001949.1), respectively (Table 2). The reaction mix consisted of HotStar Taq Mastermix with a total concentration of 6 mM MgCl_2_, 40 nM of each primer, 10 nM of the probe, and 2 µL DNA eluate per 20 µL volume. The PCR was run with an initial activation step at 95 °C for 15 min followed by 50 cycles of denaturation at 95 °C for 15 s, annealing at 60 °C for 30 s and amplification for 30 s at 72 °C, and final cooling down to 40 °C for 20 s. The limit of detection as assessed with a dilution series of the positive control plasmid was 90 copies per reaction mix (calculated for all real-time PCRs with the software SciencePrimer.com, http://scienceprimer.com/copy-number-calculator-for-realtime-pcr, last accessed 30 April 2021).

Real-time PCR 2, also targeting *Enterocytozoon bieneusi* as well as the three etiologically relevant *Encephalocytozoon* spp., was adapted from the work by Polley and colleagues as a SybrGreen-based approach [35] (Table 2). The reaction mix consisted of QuantiTect SYBR Green PCR Mastermix (Qiagen) with a total concentration of 2.5 mM MgCl_2_, 250 nM of each primer, and 2 µL DNA eluate per 20 µL volume. The PCR was run with an initial activation step at 95 °C for 15 min followed by 40 cycles of denaturation at 95 °C for 10 s, annealing at 60 °C for 20 s, and amplification for 20 s at 72 °C. After a hold for 2 min at 95 °C and a subsequent hold for 30 s at 75 °C, a melting curve was measured with the settings ramp from 75 °C to 90 °C, rising 0.6 °C per step, waiting 90 s for pre-melt conditioning at the first step, and waiting 5 s for each step afterwards. The limit of detection as assessed with a dilution series of the positive control plasmid was 87 copies per reaction mix.

The real-time PCRs 3 and 4 specifically targeted *Enterocytozoon bieneusi*. PCR 3 was adapted from Tainiuchi and colleagues [37] (Table 2). The reaction mix consisted of HotStar Taq Mastermix with a total concentration of 3 mM MgCl_2_, 160 nM of each primer, 200 nM probe, and 2 µL DNA eluate per 20 µL volume. The PCR was run with an initial activation step at 95 °C for 15 min, followed by 40 cycles of denaturation at 95 °C for 15 s, as well as by combined annealing and amplification at 58 °C for 60 s, and final cooling down to 40 °C for 10 s. The limit of detection as assessed with a dilution series of the positive control plasmid was 12 copies per reaction mix.

The oligonucleotides for PCR 4 were taken from the publication by Verweij and colleagues [33] (Table 2). The reaction mix consisted of HotStar Taq Mastermix with a total concentration of 5 mM MgCl_2_, 80 nM of each primer, 100 nM probe, and 2 µL DNA eluate per 20 µL volume. The PCR was run with an initial activation step at 95 °C for 15 min followed by 50 cycles of denaturation at 95 °C for 8 s, annealing at 60 °C for 15 s, amplification for 15 s at 72 °C, and final cooling down to 40 °C for 10 s. The limit of detection as assessed with a dilution series of the positive control plasmid was 16 copies per reaction mix. Sample inhibition control PCR targeting Phocid Herpes Virus (150 nM of each primer, 100 nM probe) as described [49] was included in a duplex real-time PCR approach together with PCR 4.

PCR 5 was also taken from the publication by Verweij and colleagues [33] (Table 2) but targeted the species *Encephalitozoon cuniculi, Encephalitozoon hellem,* and *Encephalitozoon intestinalis*. To increase the annealing temperature, the probe was slightly adapted by the inclusion of locked nucleic acids (LNAs) at three positions. The reaction mix consisted of HotStar Taq Mastermix with a total concentration of 5 mM MgCl_2_, 240 nM of each primer, 250 nM probe, and 2 µL DNA eluate per 20 µL volume. The PCR was run with an initial activation step at 95 °C for 15 min, followed by 40 cycles of denaturation at 95 °C for 15 s, annealing at 60 °C for 30 s, and amplification for 30 s at 72 °C. The limit of detection as assessed with a dilution series of the positive control plasmid was 175 copies per reaction mix.

### 4.4. Applied Non-Target-Specific PCR

To assess potential non-specific reactions with other fungal pathogens, a non-target PCR for the arbitrarily chosen, non-microsporidial fungal genus *Microsporidium* spp. (fungi with relevance for skin infections) according to Wisselink and colleagues [50] (Table 2) was added. The reaction mix consisted of HotStar Taq Mastermix with a total concentration of 1.5 mM MgCl_2_, 300 nM of each primer, 200 nM probe, and 2 µL DNA eluate per 20 µL volume. The PCR was run with an initial activation step at 95 °C for 15 min, followed by 45 cycles of denaturation at 95 °C for 15 s, as well as combined annealing and amplification at 60 °C for 60 s, and final cooling down to 40 °C for 10 s. The limit of detection as assessed with a dilution series of the positive control plasmid was 1443 copies per reaction mix.

Details of the real-time PCR assays 1–6 are summarized in Table 2 and in the Appendix A.

### 4.5. Statistical Assessment

Latent Class Analysis (LCA) was applied for the test comparison without a reference standard with perfect accuracy as described [37,38]. In detail, the results of PCR 1 vs. PCR 2 vs. PCRs 4 + 5 vs. PCRs 3 + 5 targeting both *Enterocytozoon bieneusi* and *Encephalocytozoon* spp., respectively, were compared. PCR 6 results were assessed for potential interference. By doing so, sensitivity and specificity with 0.95 confidence intervals were calculated. Cohen’s kappa indicated the agreement between the qualitative results of the real-time PCRs with the strata poor (below 0.00), slight (0.00–0.20), fair (0.21–0.40), moderate (0.41–0.60), substantial (0.61–0.80), and almost perfect (0.81–1.00), as described elsewhere [51]. Cycle threshold (Ct) values were descriptively assessed. Stata/IC 15.1 for Mac 64-bit Intel (College Station, TX, USA) was applied for the calculations.

### 4.6. Ethical Clearance

Ethical clearance was obtained from the medical association of Hamburg, Germany, (reference number: WF-011/19) without requirement for obtaining informed consent.

## 5. Conclusions

In spite of the abovementioned limitations, i.e., the use of residual samples of varying age without microscopic assessment, without detailed clinical information, and showing only a low positivity rate, the described test comparison without a reference standard with perfect accuracy suggests excellent specificity and—with one exception—good sensitivity and agreement for the assessed molecular real-time PCR assays targeting microsporidia in stool samples.

## Figures and Tables

**Table 1:** Number of positives, sensitivity, and specificity as calculated applying latent class analysis (LCA) for the different assessed microsporidia-specific real-time PCR screening assays targeting *Enterocytozoon bieneusi*, *Encephalitozoon cuniculi, Encephalitozoon hellem* and *Encephalitozoon intestinalis* as well as agreement according to Cohen’s kappa.

Assay or Assay Combination	*n*	Positives (%)	CT Value Mean (SD), Median (Min, Max)a	Sensitivity (0.95 CI)	Specificity (0.95 CI)
PCR 1	1339	87 (6.50)	24.98 (5.32), 25.70 (14.30, 34.10)	0.974 (0.899, 0.994)	0.991 (0.984, 0.995)
PCR 2	1339	47 (3.51)	29.84 (3.59), 30.10 (23.20, 35.80)	0.604 (0.492, 0.707)	1 (n.e.)
PCR 3 + 5	1339	77 (5.75)	30.22 (5.12), 30.60 (20.50, 38.30)	0.950 (0.868, 0.982)	0.998 (0.992, 0.999)
PCR 4 + 5	1339	84 (6.27)	30.79 (5.34), 31.40 (19.90, 39.50)	0.961 (0.884, 0.988)	0.993 (0.986, 0.996)
Kappa (0.95 CI)	1339	0.796 (0.742, 0.834)			

n.e. = not estimable. 0.95 CI = 95% confidence interval. Min = minimum. Max = maximum.

**Table 2:** Details of the real-time PCR assays 1–6, which were included in the test comparison without a reference standard with perfect accuracy for the diagnosis of microsporidia in stool samples. Positive control plasmid inserts are provided in Appendix A.

	PCR 1	PCR 2	PCR 3	PCR 4	PCR 5	PCR 6
Target specificity	Small subunit ribosomal RNA gene of *Enterocytozoon bieneusi*, *Encephalitozoon cuniculi*, *Encephalitozoon hellem*, and *Encephalitozoon intestinalis*	Small subunit ribosomal RNA gene of *Enterocytozoon bieneusi*, *Encephalitozoon cuniculi, Encephalitozoon hellem,* and *Encephalitozoon intestinalis*	Small subunit ribosomal RNA gene of *Enterocytozoon bieneusi*	Internal transcribed spacer (ITS) sequence of *Enterocytozoon bieneusi*	Small subunit ribosomal RNA gene of *Encephalitozoon cuniculi, Encephalitozoon hellem,* and *Encephalitozoon intestinalis*	Internal transcribed spacer (ITS) sequence of the non-target microorganism *Microsporidium* spp.
Amplicon length	394 base pairs	280 base pairs	202 base pairs	105 base pairs	227 base pairs	87 base pairs
Cycle number	50	40	40	50	40	45
Forward primer 1	5′-CACCAGGTTGATTCTGCCTGA-3′	5′-CAGGTTGATTCTGCCTGACG-3′	5′-CCAGGGTCAAGTCATTCGTT-3′	5′-TGTGTAGGCGTGAGAGTGTATCTG-3′	5′-CACCAGGTTGATTCTGCCTGAC-3′	5′-TCTTGCGCGTTAATGATCCTT-3′
Forward primer 2	5′-TCCGGAGAGGGAGCCTGAG-3′	n.a.	n.a.	n.a.	n.a.	n.a.
Reverse primer 1	5′-GCTTGCCCTCCAATTGCTTC-3′	5′-CCATCTCTCAGGCTCCCTC-3′	5′-TATTGTATTGCGCTTGCTGC-3′	5′-CATCCAACCATCACGTACCAATC-3′	5′-CTAGTTAGGCCATTACCCTAACTACCA-3′	5′-AGGTTGCGGGCGGC-3′
Reverse primer 2	5′-GACTTGCCCTCCAATCACATG-3′	n.a.	n.a.	n.a.	n.a.	n.a.
Reverse primer 3	5′-CCGACTTGCCCTCCAGTAAA-3′	n.a.	n.a.	n.a.	n.a.	n.a.
Reverse primer 4	5′-CTTGGCCTCCAATCAATCTCG-3′	n.a.	n.a.	n.a.	n.a.	n.a.
Hybridization probe *	5′-TGGCAGCAGGCGCGAAACTTGT-3′	n.a.	5′-GATGCCCTTAGATATCCTGG-3′	5′-CACTGCACCCACATCCCTCACCCTT-3′	5′-CTATCACTGAG+C+CGT+CC-3′	5′-ACGGAAGAGCTTCGGGGGCCA-3′

n.a. = not applicable. * Bases with a plus (+) in front of them are locked nucleic acid (LNA) bases.

## Data Availability

All relevant data are provided in the manuscript. Raw data can be made available upon reasonable request.

## Appendix A

**Table A1:** Sequences of the Positive Control Plasmid Inserts and Their Origins.

**Real-Time PCR Assay**	**GenBank Accession Number**	**Sequence**
PCR 1	AF023245	5′- AACACGGACCCACCAGGTTGATTCTGCCTGACGTAGATGCTAGTCTCTGAGATTAAGCCATGCATGTCAGTGAAGCCTTACGGTGGAACGGCGAACGGCTCAGTAATGTTGCGGTAATTTGGTCTCTGTGTGTAAACTAACCACGGTAACCTGTGGCTAAAAGCGGAGAATAAGGCGCAACCCTATCAGCTTGTTGGTAGTGTAAAGGACTACCAAGGCCATGACGGGTAACGGGAAATCAGGGTTTGATTCCGGAGAGGGAGCCTGAGAGATGGCTCCCACGTCCAAGGACGGCAGCAGGCGCGAAACTTGTCCACTCCTTACGGGGGAGACAGTCATGAGACGTGAGTATAAGACCTGAGTGTAAAGACCTTAGGGTGAAGCAATTGGAGGGCAAGCTTTGGTGCCAGCAGC-3′
PCR 2	KC513629.1	5′- GACGTAGTAGCCATCTCTCAGGCTCCCTCTCCGGAACCAAACCCTGATCCCCCGTATCCCGTCTGCGCCTAGTTAGGCCATTACCCTAACTACCAGCTGATAGGCCCACAACTTACTTGCCAACCCCCACAGGGGCAGACCACTATCTGCAGTTTCCCGCAGCTACTGCTCATCCCGCAAACAAATCATCGTGCTATCACTGAGCCGTCCGCTAATCCCCCACAAAGAGTTCACAAGCATGCATGGCTTAGCCCCAGAGAATAGCATCCACGTCAGGCAGAATCAACCTGATGCCCCACA-3′
PCR 3	AF024657	5′- GGAAAACTTACCAGGGTCAAGTCATTCGTTGATCGAATACGTGAGAATGGCAGGAGTGGTGCATGGCCGTTGGAAATTGATGGGGCGACCTTTAGCTTAAATGCTTAAACCAGTGAGACCTCCTTGACAGGTGTTCTGTAACACAGGAGGGTGGAGGCTATAACAGGTCCGTGATGCCCTTAGATATCCTGGGCAGCAAGCGCAATACAATATCTCTTCAGT-3′
PCR 4	AF023245	5′- GGTGCGGTGGTGTGTGCAGGCGTGAGAGTGTATCTGCAAGTGTGAGGGATGTGGGTGCAGCGAGTTAGAGGTGGTTCCATGTGGAATAGTGGGATTGGTACGTGATGGTTGGATGGGGGAATGAT-3′
PCR 5	U09929	5′- AGGATCATAACACCAGGTTGATTCTGCCTGACGTGGATGCTATTCTCTGGGACTAAGCCATGCATGTTGATGAACCTTGTGGGGGATTGACGGACGGCTCAGTGATAGTACGATGATTTGGTTGGCGGGAGAGCTGTAACTGCGGGAAACTGCAGGTAGGGGGCTAGGAGTGTTTTTGACACGAGCCAAGTAAGTTGTAGGCCTATCAGCTGGTAGTTAGGGTAATGGCCTAACTAGGCGGAGACGG-3′
PCR 6	GU291265	5′- GAACCTGCGGAAGGATCATTAACGCGCAAGAGGTCGAAGTTGGCCCCCGAAGCTCTTCCGTCTCCCCCCCGGGCCTCCCGGGGAGGTTGCGGGCGGCGAGGGGTGCC-3′
