# Correction: Tanida et al. Comparative Assessment of In-House Real-Time PCRs Targeting Enteric Disease-Associated Microsporidia in Human Stool Samples. *Pathogens* 2021, *10*, 656

**DOI:** 10.3390/pathogens11020256

**Published:** 2022-02-17

**Authors:** Konstantin Tanida, Andreas Hahn, Kirsten Alexandra Eberhardt, Egbert Tannich, Olfert Landt, Simone Kann, Torsten Feldt, Fred Stephen Sarfo, Veronica Di Cristanziano, Hagen Frickmann, Ulrike Loderstädt

**Affiliations:** 1 Department of Microbiology and Hospital Hygiene, Bundeswehr Hospital Hamburg, 20359 Hamburg, Germany; konstantintanida@bundeswehr.org (K.T.); frickmann@bnitm.de (H.F.); 2 Department of Medical Microbiology, Virology and Hygiene, University Medicine Rostock, 18057 Rostock, Germany; hahn.andreas@me.com; 3 Department of Tropical Medicine, Bernhard Nocht Institute for Tropical Medicine & I. Department of Medicine, University Medical Center Hamburg-Eppendorf, 20251 Hamburg, Germany; k.eberhardt@bnitm.de; 4 Institute for Transfusion Medicine, University Medical Center Hamburg-Eppendorf, 20251 Hamburg, Germany; 5 Bernhard Nocht Institute for Tropical Medicine Hamburg, 20359 Hamburg, Germany; tannich@bnitm.de; 6 National Reference Centre for Tropical Pathogens, 20359 Hamburg, Germany; 7 TIB MolBiol, 12103 Berlin, Germany; oLandt@tib-molbiol.de; 8 Medical Mission Institute, 97074 Würzburg, Germany; simone_kann@hotmail.com; 9 Department of Gastroenterology, Hepatology and Infectious Diseases, University Medical Center Düsseldorf, 40225 Düsseldorf, Germany; torsten.feldt@med.uni-duesseldorf.de; 10 Department of Medicine, Kwame Nkrumah University of Science and Technology, Kumasi, Ghana; stephensarfo78@gmail.com; 11 Institute of Virology, Faculty of Medicine and University Hospital of Cologne, University of Cologne, 50935 Cologne, Germany; veronica.di-cristanziano@uk-koeln.de; 12 Department of Hospital Hygiene & Infectious Diseases, University Medicine Göttingen, 37075 Göttingen, Germany

In the original publication [1], there was a mistake in Table 2 as published. All oligonucleotides of PCR 1 in Table 2 had erroneously been printed in reverse-complement style. The corrected Table 2 appears below. The authors apologize for any inconvenience caused and state that the scientific conclusions are unaffected. The original publication has also been updated.

## Figures and Tables

**Table 2:** Details of the real-time PCR assays 1–6, which were included in the test comparison without a reference standard with perfect accuracy for the diagnosis of microsporidia in stool samples. Positive control plasmid inserts are provided in Appendix A.

	PCR 1	PCR 2	PCR 3	PCR 4	PCR 5	PCR 6
Target specificity	Small subunit ribosomal RNA gene of *Enterocytozoon bieneusi*, *Encephalitozoon cuniculi*, *Encephalitozoon hellem*, and *Encephalitozoon intestinalis*	Small subunit ribosomal RNA gene of *Enterocytozoon bieneusi*, *Encephalitozoon cuniculi, Encephalitozoon hellem,* and *Encephalitozoon intestinalis*	Small subunit ribosomal RNA gene of *Enterocytozoon bieneusi*	Internal transcribed spacer (ITS) sequence of *Enterocytozoon bieneusi*	Small subunit ribosomal RNA gene of *Encephalitozoon cuniculi, Encephalitozoon hellem,* and *Encephalitozoon intestinalis*	Internal transcribed spacer (ITS) sequence of the non-target microorganism *Microsporidium* spp.
Amplicon length	394 base pairs	280 base pairs	202 base pairs	105 base pairs	227 base pairs	87 base pairs
Cycle number	50	40	40	50	40	45
Forward primer 1	5′-CACCAGGTTGATTCTGCCTGA-3′	5′-CAGGTTGATTCTGCCTGACG-3′	5′-CCAGGGTCAAGTCATTCGTT-3′	5′-TGTGTAGGCGTGAGAGTGTATCTG-3′	5′-CACCAGGTTGATTCTGCCTGAC-3′	5′-TCTTGCGCGTTAATGATCCTT-3′
Forward primer 2	5′-TCCGGAGAGGGAGCCTGAG-3′	n.a.	n.a.	n.a.	n.a.	n.a.
Reverse primer 1	5′-GCTTGCCCTCCAATTGCTTC-3′	5′-CCATCTCTCAGGCTCCCTC-3′	5′-TATTGTATTGCGCTTGCTGC-3′	5′-CATCCAACCATCACGTACCAATC-3′	5′-CTAGTTAGGCCATTACCCTAACTACCA-3′	5′-AGGTTGCGGGCGGC-3′
Reverse primer 2	5′-GACTTGCCCTCCAATCACATG-3′	n.a.	n.a.	n.a.	n.a.	n.a.
Reverse primer 3	5′-CCGACTTGCCCTCCAGTAAA-3′	n.a.	n.a.	n.a.	n.a.	n.a.
Reverse primer 4	5′-CTTGGCCTCCAATCAATCTCG-3′	n.a.	n.a.	n.a.	n.a.	n.a.
Hybridization probe *	5′-TGGCAGCAGGCGCGAAACTTGT-3′	n.a.	5′-GATGCCCTTAGATATCCTGG-3′	5′-CACTGCACCCACATCCCTCACCCTT-3′	5′-CTATCACTGAG+C+CGT+CC-3′	5′-ACGGAAGAGCTTCGGGGGCCA-3′

n.a. = not applicable. * Bases with a plus (+) in front of them are locked nucleic acid (LNA) bases.
